# Absent Septum Pellucidum in Fetal Development: Diagnostic Challenges, Associated Anomalies, and Prognostic Uncertainty—A Structured Narrative Review

**DOI:** 10.3390/jcm15134889

**Published:** 2026-06-23

**Authors:** Agnieszka Helena Czapska, Beata Rebizant, Katarzyna Kosińska-Kaczyńska

**Affiliations:** Department of Obstetrics, Perinatology and Neonatology, Center of Postgraduate Medical Education, Cegłowska St. 80, 01-809 Warsaw, Poland; ahczapska@gmail.com (A.H.C.); beata.rebizant@gmail.com (B.R.)

**Keywords:** absent septum pellucidum, cavum septi pellucidi, fetal MRI, prenatal ultrasound, septo-optic dysplasia, corpus callosum, ventriculomegaly, prenatal diagnosis, neurodevelopmental outcome, structured narrative review

## Abstract

**Background/Objectives**: Absent septum pellucidum (ASP) is a rare fetal midline brain finding that may occur in isolation or alongside broader central nervous system (CNS) malformations, genetic disorders, or septo-optic dysplasia (SOD). Accurate prenatal diagnosis and counseling remain challenging because apparently isolated ASP may be reclassified following fetal magnetic resonance imaging (MRI), postnatal neuroimaging, or specialist assessment. This structured narrative review aimed to synthesize current evidence on prenatal imaging findings, associated anomalies, genetic evaluation, and postnatal outcomes in fetuses with ASP. **Methods**: This structured narrative review used PRISMA-informed reporting. PubMed and Google Scholar were searched for full-text English-language studies published from 2014 through the updated search date (8 June 2026). Data on gestational age at diagnosis, imaging classification, associated anomalies, genetic testing, postnatal assessment, and neurodevelopmental, ophthalmological, and endocrine outcomes were extracted. Study methodological quality was appraised using Joanna Briggs Institute tools. **Results**: Seven studies comprising 342 fetal ASP cases were included. Of these, 94 cases (27.5%) were classified as isolated ASP prenatally, but only 57 remained isolated postnatally when follow-up data were available. SOD was confirmed after birth in 11 of 94 (11.7%) fetuses with prenatally isolated ASP. As definitions, imaging protocols, genetic testing strategies, and follow-up duration differed substantially across studies, these pooled values are descriptive observations rather than formal quantitative estimates. **Conclusions**: ASP is a heterogeneous prenatal finding. The prognosis is most favorable when ASP remains isolated following a detailed prenatal and postnatal evaluation. Multidisciplinary follow-up involving fetal medicine, neuroradiology, genetics, ophthalmology, endocrinology, and neurology is essential for risk stratification and counseling.

## 1. Introduction

The septum pellucidum (SP) is a midline cerebral structure located between the bodies of the lateral ventricles and the medial portions of the frontal horns. It is bounded inferiorly by the fornix and superiorly by the corpus callosum (CC). The SP consists of two thin leaflets, and the fluid-filled space between them is known as the cavum septi pellucidi (CSP). The SP contains white matter fibers and is considered relevant to the neurodevelopmental organization of limbic pathways [1,2,3]. Absence of the septum pellucidum (ASP) may therefore represent an isolated midline variant or a marker of wider cerebral malformation, genetic disease, or syndromic disease [1].

The CSP is usually seen as a rectangular or triangular space in the anterior fetal brain, between the anterior horns of the lateral ventricles. It should be visible on prenatal ultrasound (US) between approximately 17 and 37 weeks of gestation. In axial and coronal planes, its width should not exceed 10 mm at any gestational age. The anterior part of the cavity is known as the CSP, whereas the posterior extension behind the foramen of Monro is known as the cavum vergae. Physiological closure starts posteriorly and progresses anteriorly near the end of gestation [1].

In ASP, the septal leaflets that form the walls of the CSP are partially or completely absent, leading to communication between the frontal horns. ASP is rare, with an estimated incidence of two to three per 100,000 individuals, and it may be complete or partial, or occur in isolation or as part of a complex malformation. Complex ASP is accompanied by other CNS or extracranial malformations [1,4]. The existence and frequency of truly isolated ASP remain debated. Sundarakumar et al. suggested that isolated ASP without associated abnormalities is rare and difficult to diagnose [4]. Conversely, Nagaraj et al. reported that 75% of fetuses with an absent CSP on fetal MRI had no clinical, endocrine, or ophthalmological abnormalities after birth, suggesting that isolated septal deficiency may be more prevalent than previously thought [5].

Several diagnostic algorithms have been proposed for fetuses with non-visualized CSP or suspected ASP [2,3,5,6,7]. A practical approach begins by determining whether the frontal horns communicate. If they do, holoprosencephaly (HPE), including lobar, semilobar, and alobar forms, should be considered, particularly if cortical or basal cleavage is abnormal or if there are facial anomalies [5,6]. If frontal horn communication is absent and normal cleavage is demonstrated, the corpus callosum (CC) should be assessed. A visible CC with a CSP width below 10 mm indicates a normal CSP, whereas a wide CSP suggests the presence of a CSP cyst or other variants [5,7]. If the CC is not visualized, agenesis or dysgenesis of the corpus callosum (ACC) should be suspected and investigated further [5,6,7].

The differential diagnosis also requires assessment for severe ventriculomegaly (VMG), acquired ASP secondary to hydrocephalus, Chiari II malformation, cephalocele, hemorrhage, infection, trauma, hydranencephaly, schizencephaly, and SOD [5,6]. Apparently isolated ASP can only be diagnosed after detailed exclusion of associated abnormalities. When another anomaly is present, the prognosis is largely determined by the type and severity of that anomaly [5,6].

Distinguishing ASP from lobar holoprosencephaly (HPE) is particularly important. In lobar HPE, the frontal horns are fused, the anterior interhemispheric fissure may be absent, and the fornix columns are fused. Facial anomalies are also common. In ASP, however, the frontal horns are separated, the interhemispheric fissure is visible, and the fornix columns are typically separated or partially preserved. Facial anatomy is also usually normal [8,9]. The prognosis for lobar HPE can range from survival with near-normal function to psychomotor impairment and neurological deficits, with more severe facial anomalies correlating with worse survival [8,10].

CC anomalies are among the most frequent CNS malformations. Prenatal diagnosis begins with indirect US signs in the axial transventricular plane, including an absent or abnormal CSP, separated and thin anterior horns of the lateral ventricles, a widened interhemispheric space, an elevated third ventricle between the lateral ventricles, and colpocephaly [11,12]. Sagittal views may demonstrate an abnormal pericallosal artery and a radial sulcal pattern, while coronal views may reveal the characteristic “Viking helmet” appearance, particularly in cases of complete ACC [11,12]. The prognosis in cases of complete ACC, partial ACC, and CC dysgenesis is primarily determined by associated malformations [12,13,14].

SOD is one of the most clinically important conditions to consider because it cannot be completely excluded prenatally. It is a heterogeneous syndrome classically defined by ASP, optic nerve hypoplasia, and pituitary abnormalities, with hypothalamic–pituitary dysfunction occurring in up to 70% of cases [15]. Prenatal biochemical assessments, such as measuring maternal estriol or fetal serum levels of growth hormone, adrenocorticotropic hormone, prolactin, and thyroid-stimulating hormone, may suggest endocrine dysfunction. However, hormonal profiles are inconclusive because endocrine deficits may not become apparent until later in infancy [16]. SOD Plus refers to SOD accompanied by cortical malformations, most often schizencephaly [17].

Visualization and measurement of the fetal optic chiasm (OC) using two-dimensional transabdominal US are feasible and reproducible. Chiasmatic decussation is preferred over optic nerve measurement because optic nerve size is more variable [18]. While a normal OC does not exclude SOD, it appears to reduce the residual risk; conversely, abnormal OC, optic tract, or optic nerve findings heighten suspicion [19,20]. Even postnatal MRI scans may appear normal in some cases of SOD; a definitive diagnosis of optic pathway involvement requires a thorough ophthalmological examination, and endocrine abnormalities may develop over time [19,20,21].

This structured narrative review aimed to summarize the current evidence on prenatal imaging of ASP, associated anomalies, and postnatal outcomes, with a focus on diagnostic challenges, prognostic factors, genetic evaluation, risk of bias, and the need for longitudinal, multidisciplinary follow-up.

## 2. Materials and Methods

This structured narrative review used predefined eligibility criteria, a reproducible search strategy, and PRISMA-informed reporting to evaluate studies describing the prenatal diagnosis of ASP and its postnatal or perinatal outcomes [22]. The term structured narrative review is used consistently throughout this manuscript; PRISMA-informed refers only to reporting the search and selection process and not to the review design. The PRISMA checklist, full electronic search strategy, detailed study characteristics, and methodological quality assessment are provided as Supplementary Material. The review was not preregistered. Because the search was limited to PubMed and Google Scholar, and because fully independent duplicate screening and extraction were not performed at all stages, the findings should be interpreted as a structured descriptive synthesis rather than as a meta-analysis.

The initial literature search was performed on 5 November 2024 and updated on 8 June 2026, using the same databases, search strings, language restrictions, and eligibility criteria. PubMed (National Library of Medicine, Bethesda, MD, USA; https://pubmed.ncbi.nlm.nih.gov/; accessed on 8 June 2026) and Google Scholar (Google LLC, Mountain View, CA, USA; https://scholar.google.com/; accessed on 8 June 2026) were searched for English-language full-text studies published from 2014 through the updated search date. To improve retrieval of potentially relevant studies, the reference lists of eligible studies and relevant reviews were manually screened. The search strategy combined free-text terms and, where available, Medical Subject Headings (MeSH): “absent septum pellucidum” AND “fetal MRI”; “absent septum pellucidum” AND “prenatal ultrasound”; “absent septum pellucidum” AND “holoprosencephaly”; and “absent septum pellucidum” AND “septo-optic dysplasia”. The complete electronic search strategy is provided in Supplementary Table S2. The final search verification confirmed that all eligible primary prenatal ASP outcome cohorts identified through 8 June 2026 had been considered and cited where appropriate; recent narrative or practical recommendation papers identified during the update were cited only for contextual discussion and were not extracted as primary outcome cohorts. No equipment, machines, chemicals, or dedicated systematic-review management software were used.

**Figure 1:**
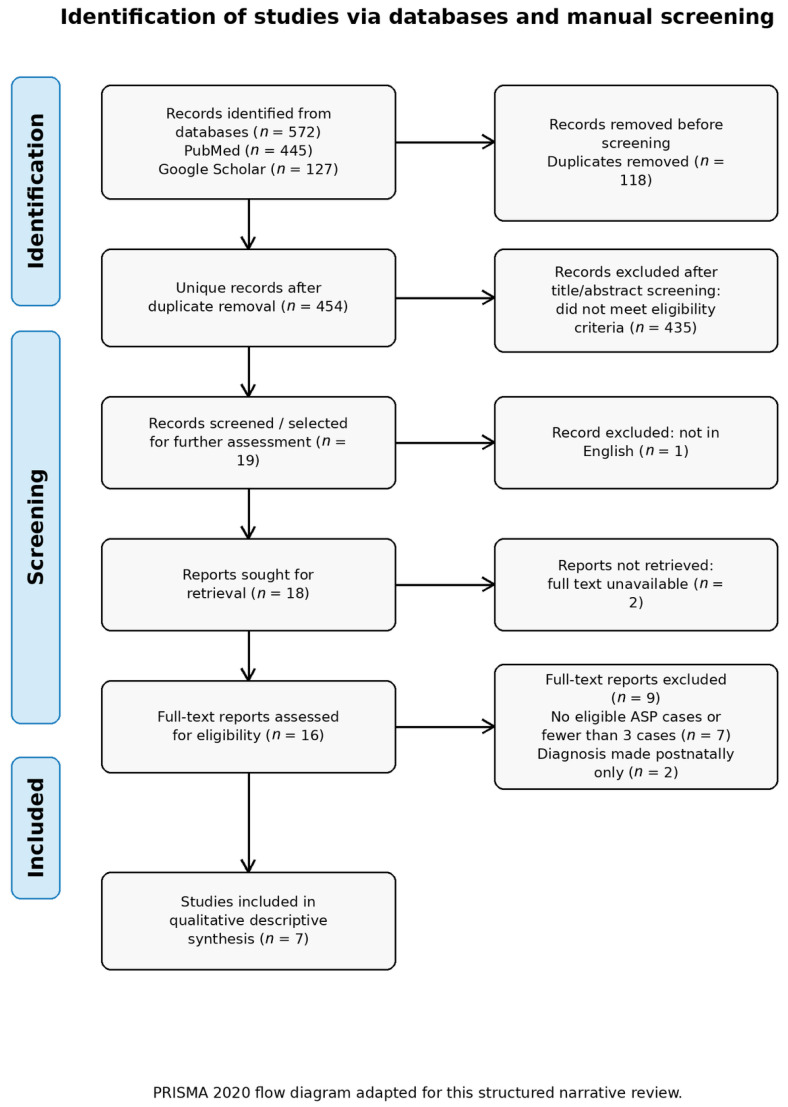
PRISMA 2020 flow diagram for study selection in this structured narrative review. ASP, absent septum pellucidum.

Titles and abstracts were screened by one reviewer. Full-text eligibility decisions and extracted data were subsequently verified by a second reviewer for accuracy, and uncertainties were resolved through discussion with the senior author. Extracted variables included study design, sample size, prenatal imaging findings, genetic testing modality and results, postnatal imaging, ophthalmological and endocrine assessments, age and duration of follow-up when reported, loss to follow-up, and neurodevelopmental outcomes. As initial title and abstract screening was not performed independently in duplicate, selection bias cannot be fully excluded. The study-selection process is summarized in Figure 1.

Eligible studies were full-text retrospective or cohort studies written in English that used prenatal US and/or MRI to diagnose ASP, investigated associated CNS or extracranial anomalies and/or genetic findings, and reported perinatal, ophthalmological, endocrine, or neurodevelopmental outcomes. Studies were excluded if they were case reports or series with fewer than three subjects, conference abstracts, reviews without primary clinical data, reports with a postnatal diagnosis only, articles without full-text access, or articles published in languages other than English.

For the purposes of this review, isolated ASP was defined as the absence of the septum pellucidum without any additional major CNS or extracranial anomalies identified on prenatal imaging. Cases with associated corpus callosum abnormalities, HPE, cortical malformations, severe VMG, posterior fossa anomalies, extracranial malformations, or suspected syndromic disease were classified as complex ASP. As definitions varied among the included studies, cases with mild VMG were recorded according to the classification used in the original publication and interpreted cautiously. The methodological quality of the studies was assessed using the Joanna Briggs Institute critical appraisal tools for cohort and case series studies, as appropriate. Potential patient overlap was assessed by comparing study period, country, center, authorship, and cohort description. No definite duplication was identified; however, limited overlap cannot be fully excluded, as several reports were retrospective referral-center cohorts. The anticipated principal review-level limitations were the small number of eligible studies, heterogeneity in the definition of isolated ASP, variable inclusion of mild VMG within isolated ASP cohorts, inconsistent genetic work-up, variable postnatal imaging and specialist assessment, and short or incomplete follow-up in several studies.

## 3. Results

The original database search identified 572 records, including 445 from PubMed and 127 from Google Scholar. After removing 118 duplicate records, 454 unique records remained. Of these, 435 were excluded during title and abstract screening because they did not meet the eligibility criteria, leaving 19 records for further assessment. One record was excluded because it was not published in English. Eighteen reports were sought for full-text retrieval, of which two were excluded because the full text was unavailable. Of the 16 full-text reports assessed for eligibility, nine were excluded because they described no eligible ASP cases, included fewer than three cases, or reported diagnosis only after birth. The updated search performed on 8 June 2026 did not identify any additional eligible primary prenatal ASP outcome cohorts for inclusion, confirming that the eligible primary cohorts available through the final search date were represented by the seven studies included in the structured descriptive synthesis. These seven studies were therefore retained as the complete set of eligible primary outcome cohorts for this review.

The included studies comprised 342 fetuses with ASP diagnosed in tertiary referral settings in Italy, France, Spain, Portugal, Israel, Chile, Colombia, the United States, and Canada. Prenatal diagnosis was based on US, MRI, or both. One included publication contained both an original cohort and a meta-analysis. For the purposes of the present descriptive synthesis, only the newly reported cohort cases from that publication were extracted and included. Data derived from the meta-analysis component were not entered as primary cases in order to avoid duplicating patients who were already represented in other included studies. While no definite patient overlap was identified across the included cohorts, limited overlap cannot be fully excluded, as recruitment details were incompletely reported in some retrospective studies. Postnatal follow-up was not available for all fetuses, and when available, it often consisted of short observation periods.

Methodological quality and risk of bias. The included studies were mainly retrospective, referral-center cohorts or small case series and were judged to have low-to-moderate, moderate, or moderate-to-high risk of bias. The most frequent limitations were small sample size, incomplete postnatal confirmation, heterogeneous imaging protocols, inconsistent genetic testing, variable follow-up duration, and potential outcome bias due to termination of pregnancy or loss to follow-up. Studies with systematic postnatal ophthalmological and endocrine assessments provided stronger evidence for SOD-related outcomes; however, generalizability remained limited because of small numbers and referral-center enrichment. The detailed JBI assessment is provided in Supplementary Table S3. The characteristics and main findings of the included studies are summarized in Table 1.

### 3.1. Timing and Imaging Diagnosis

Across the included studies, the mean gestational age at prenatal diagnosis was approximately 26 + 2 weeks. US was the first-line screening modality, but it had limited sensitivity for subtle or coexisting CNS abnormalities, including cortical malformations and optic pathway anomalies. Consequently, fetuses initially classified as having isolated ASP were sometimes reclassified after fetal MRI or postnatal assessment.

Fetal MRI was used to clarify uncertain US findings and to identify associated abnormalities that were not readily visible on ultrasound. It was particularly useful for detecting cortical abnormalities, callosal anomalies, septal remnants, fused forniceal columns, and optic pathway abnormalities. However, neither fetal US nor MRI could definitively rule out SOD during the prenatal period.

### 3.2. Coexisting Abnormalities

A major finding across the reviewed literature was that apparently isolated ASP was frequently reclassified as complex ASP following additional imaging or postnatal evaluation. Associated abnormalities included ACC or CC dysgenesis, severe VMG, aqueductal stenosis, schizencephaly, polymicrogyria, cerebellar hypoplasia, heterotopia, Dandy-Walker malformation, microcephaly, and extracranial anomalies. Complex ASP was consistently associated with a higher risk of adverse neurodevelopmental outcomes, seizures, hydrocephalus, visual impairment, and endocrine dysfunction.

Of the 94 fetuses classified prenatally as having isolated ASP, 57 (60.6%) remained isolated postnatally when follow-up information was available. SOD was diagnosed postnatally in 11 of 94 (11.7%) fetuses with prenatally isolated ASP. However, these pooled values should be interpreted strictly as descriptive observations rather than formal meta-analytic estimates, given that the definitions of isolated ASP, the inclusion of mild VMG, genetic testing strategies, postnatal assessment, and follow-up duration varied across studies. This residual risk remains central to prenatal counseling because SOD cannot be reliably excluded before birth, even when fetal optic pathway imaging is reassuring.

### 3.3. Genetic Findings

Genetic testing was performed inconsistently and reported incompletely across the included studies; the reported genetic work-up and diagnostic yield are summarized in Table 2. Reported modalities included karyotyping, chromosomal microarray, and unspecified prenatal genetic or screening tests. No study reported a standardized exome sequencing strategy for all ASP cases. Most tested cases of apparently isolated ASP had normal results, but this observation should be interpreted cautiously because the numbers tested, reasons for testing, and testing methods differed substantially between studies. Clinically relevant chromosomal abnormalities, microdeletions, FOXG1 syndrome, and variants of uncertain significance were mainly reported in complex or selected cases. Therefore, the diagnostic yield of each testing approach could not be reliably pooled.

### 3.4. Perinatal and Postnatal Outcomes

TOP was more frequent in complex ASP cases or when SOD was strongly suspected. For example, Borkowski-Tillman et al. reported nine terminations among complex cases with CNS findings, and Shinar et al. reported four terminations in fetuses with isolated ASP suspected of SOD [21,25]. Stillbirth was reported in a small number of complex or SOD-suspected cases [21].

Postnatal evaluation was crucial for final classification. Ophthalmological and endocrine assessments detected optic nerve hypoplasia and pituitary dysfunction in some infants whose prenatal imaging had suggested isolated ASP. Although normal prenatal OC measurements reduced the risk of later SOD-related findings, they did not eliminate it [20,26,27].

Long-term outcomes differed sharply between postnatally isolated ASP and postnatally complex ASP, but interpretation of outcomes is limited by heterogeneous follow-up. Follow-up data on neuromotor and cognitive development were available for 103 infants, including 55 with postnatally isolated ASP and 48 with postnatally complex ASP. Of the children with postnatally isolated ASP, 47/55 (85.5%) had normal development, whereas 8/55 (14.5%) had reported abnormalities such as communication delay, gross motor delay, hypertonia, abnormal vision, seizures, or need for therapy. In postnatally complex ASP, only 10/48 children (20.8%) had normal development, while 38/48 (79.2%) had neurodevelopmental or neurological impairment. These values are descriptive observations only and should not be interpreted as precise prognostic estimates, as follow-up age, duration, assessment tools, and loss to follow-up varied across studies. Neuromotor and cognitive outcomes are summarized in Table 3.

Follow-up details differed substantially between cohorts. Vawter-Lee et al. provided the most standardized assessment, involving postnatal MRI, ophthalmological evaluation, endocrine evaluation, and developmental screening using the Ages and Stages Questionnaire (ASQ-3) or the Parents’ Evaluation of Developmental Status (PEDS) at 8 months to 6 years of age. Borkowski-Tillman et al. reported follow-up for all 14 live-born cases of isolated ASP, with a mean follow-up duration of 45 months (range, 3–84 months). Follow-up in complex cases was shorter and less complete. Di Pasquo et al. reported a median postnatal follow-up period of 36 months (range, 12–60 months) for the original cohort. Neurological, ophthalmological, and endocrine information was obtained from medical records or telephone questionnaires. Pickup et al. reported postnatal follow-up in 22 of 33 live-born infants, with an average interval between the first and most recent visits of 520 ± 515 days, and postnatal MRI for most children under observation. Pilliod et al. conducted postnatal imaging in ten cases, as well as pituitary and ophthalmological assessments in surviving neonates. However, long-term clinical follow-up was incomplete and not standardized. Viñals et al. reported complete follow-up for all eight ASP cases, ranging from 6 months to 7 years, with postnatal ophthalmological assessment performed in all cases. These differences limit direct comparison across studies and suggest that developmental proportions should be interpreted as descriptive observations only.

**Table 3:** Neuromotor and cognitive development in children with postnatally isolated and postnatally complex ASP. Follow-up methods, age at assessment, and loss to follow-up varied across studies; therefore, values are descriptive only. Detailed study-level follow-up characteristics are summarized in Supplementary Table S5.

Outcome	Postnatally Isolated ASP (*n* = 55)	Postnatally Complex ASP (*n* = 48)
Global developmental delay	0	3
Developmental delay: communication/language	1	8
Gross motor delay	2	6
Fine motor delay	0	3
Required physical, occupational, or speech therapy	2	4
Hypertonia	1	0
Hypotonia	0	2
Abnormal vision	1	2
Abnormal hearing	0	2
Abnormal feeding	0	3
Hydrocephalus	0	8
Seizures	1	5
Normal development	47	10

ASP, absent septum pellucidum. Some children had more than one outcome category. Postnatal outcomes and SOD risk in fetuses with prenatally isolated ASP are summarized in Table 4.

**Table 4:** Postnatal outcomes and SOD risk in fetuses with prenatally isolated ASP.

Study	Prenatally Isolated ASP	Postnatally Isolated ASP	SOD from Prenatally Isolated ASP Group
Pickup et al. [23]	17	5	1
Vawter-Lee et al. [24]	8	4	2
Borkowski-Tillman et al. [25]	17	14	0
Shinar et al. [21]	18	6	5
Pilliod et al. [26]	14	9	2
Di Pasquo et al. [20]	15	14	1
Viñals et al. [27]	5	5	0
Total	94 (100%)	57 (60.6%)	11 (11.7%)

**Figure 2:**
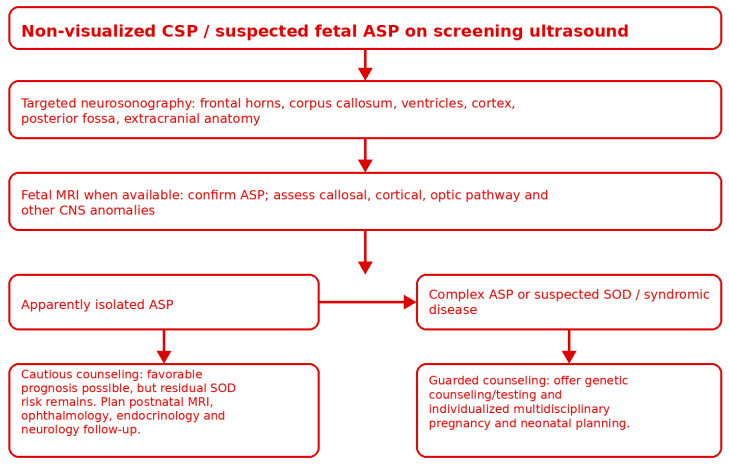
Proposed diagnostic and counseling pathway for suspected prenatal absent septum pellucidum. ASP, absent septum pellucidum; CNS, central nervous system; CSP, cavum septi pellucidi; MRI, magnetic resonance imaging; SOD, septo-optic dysplasia.

ASP, absent septum pellucidum; SOD, septo-optic dysplasia. A proposed diagnostic and counseling pathway for suspected prenatal ASP is shown in Figure 2.

## 4. Discussion

This structured narrative review confirms that ASP is diagnostically and prognostically heterogeneous. The main challenge in prenatal diagnosis is to distinguish truly isolated ASP from complex ASP and from early or subtle manifestations of SOD. Fetal position, limited acoustic windows, gestational age, and incomplete visualization of the OC, optic tracts, optic nerves, and cortical development may obscure clinically important findings. Even when prenatal imaging suggests isolated ASP, postnatal SOD or other neurological abnormalities may emerge, highlighting the importance of thorough genetic evaluation and long-term monitoring.

US remains the first-line modality for fetal brain assessment because it is widely available, noninvasive, and suitable for real-time screening. However, ultrasound alone may underestimate subtle findings such as cortical malformations, thin septal remnants, fused forniceal columns, mild optic pathway abnormalities, and partial callosal dysgenesis. Across the included studies, fetal MRI refined the diagnosis in selected cases and sometimes revealed additional abnormalities that altered counseling and follow-up plans [20,21,23,24,25,26,27]. Recent literature focusing on fetal MRI also supports a systematic, algorithmic approach when the CSP is not visualized. This approach includes assessing the corpus callosum, cortical development, ventricles, optic pathway structures, and other midline abnormalities [28]. Nevertheless, fetal MRI also has important limitations and cannot definitively exclude SOD, particularly because visual and endocrine manifestations may only become apparent after birth [29].

The prognosis of ASP depends strongly on final classification. Children with postnatally isolated ASP generally had favorable outcomes, with normal development reported in 85.5% of cases with follow-up in this review. Mild developmental delay, abnormal tone, visual problems, or seizures occurred in a minority of cases. These findings support cautious reassurance when detailed prenatal imaging, genetic testing, and postnatal assessment confirm isolated ASP, while emphasizing the need for follow-up.

SOD presents the main counseling challenge in cases of apparently isolated ASP. In this review, SOD was diagnosed after birth in 11.7% of fetuses initially considered to have isolated ASP. This percentage should be interpreted descriptively, as the contributing studies differed in their inclusion criteria and follow-up intensity. Di Pasquo et al. reported a residual SOD risk of approximately 19% in cases of apparently isolated ASP, a risk that was reduced when prenatal OC, optic tract, and optic nerve evaluation was normal [20]. Recent practical recommendations for prenatally diagnosed ASP and SOD emphasize that a definitive SOD diagnosis requires postnatal evaluation, including ophthalmological and endocrine assessments. Counseling should address uncertainty surrounding visual, endocrine, and neurodevelopmental outcomes [29]. SOD-related morbidity may include visual impairment ranging from mild deficits to blindness, as well as endocrine dysfunction requiring lifelong hormonal surveillance and replacement therapy. As endocrine deficits can evolve over time, a normal neonatal assessment does not completely rule out the possibility of later pituitary dysfunction.

Complex ASP carries a substantially higher risk of an adverse neurodevelopmental outcome. When ASP is associated with ACC, VMG, HPE, cortical malformations, cerebellar abnormalities, or extracranial anomalies, outcomes are more often characterized by developmental delay, epilepsy, hydrocephalus, feeding difficulties, hearing impairment, visual impairment, and reduced quality of life. In these cases, the prognosis is determined less by the absence of the SP itself and more by the associated cerebral and systemic abnormalities.

Genetic testing is an important component of the evaluation process, but the evidence base remains incomplete. While most tested cases of isolated ASP had normal results, the available data do not allow for reliable calculation of modality-specific diagnostic yield. Chromosomal microarray analysis can identify pathogenic copy-number variants that conventional karyotyping cannot detect, and it should be considered, especially when ASP is associated with additional CNS or extracranial anomalies. In cases of apparently isolated ASP, the diagnostic yield appears lower. However, normal genetic testing does not eliminate the risk of monogenic disease or the later emergence of neurodevelopmental abnormalities. Exome sequencing may be considered in selected complex cases, especially when chromosomal microarray analysis is normal and additional structural anomalies are present. However, the evidence base for routine exome sequencing in cases of isolated ASP remains limited. Genetic results should therefore be interpreted alongside detailed imaging and family counseling.

Isolated ASP shares several counseling challenges with other apparently isolated fetal midline CNS anomalies, particularly isolated corpus callosum abnormalities. In both conditions, the prognosis depends heavily on whether the finding remains isolated following advanced fetal imaging, genetic evaluation, and postnatal assessment. However, ASP differs clinically in that the major residual uncertainty concerns not only occult cortical or callosal malformation, but also SOD, which may not be definitively diagnosed until after birth. Therefore, postnatal ophthalmological and endocrine surveillance is specifically required for apparently isolated ASP, even when fetal MRI and prenatal optic pathway assessment are reassuring.

### Strengths and Limitations

A strength of this review is its descriptive synthesis of 342 fetal ASP cases, including both isolated and complex presentations, as well as its focused analysis of developmental outcomes where follow-up was available. The review also highlights clinically relevant discrepancies between prenatal and postnatal classification, quantifies the residual risk of SOD among fetuses initially classified as having isolated ASP, and includes a structured methodological quality assessment of the included studies.

The main limitations arise from the literature included in the review and from the review process itself. Sample sizes were small in several studies, imaging protocols were heterogeneous, genetic testing was inconsistently performed, and follow-up duration varied substantially. The definitions of isolated ASP differed across studies, particularly with regard to whether mild VMG was permitted. This variability has likely contributed to differences in postnatal reclassification rates, SOD risk, and developmental outcomes. TOP in complex ASP cases also limits complete outcome assessment. These factors restrict generalizability and preclude formal meta-analysis of most outcomes. At the review level, the search was limited to PubMed and Google Scholar because the authors did not have institutional access to Embase, Scopus, or Web of Science. Therefore, relevant studies indexed only in subscription databases may have been missed. The review was not prospectively registered in PROSPERO or another prospective review registry, and initial title and abstract screening was not performed independently in duplicate. Full-text eligibility decisions and extracted data were verified by a second reviewer, but selection bias cannot be fully excluded. Manual screening of reference lists and an updated search performed on 8 June 2026 were used to partially mitigate these limitations.

## 5. Conclusions

ASP is a heterogeneous prenatal finding with outcomes ranging from normal development to severe neurological, visual, and endocrine impairment. The prognosis is most favorable when ASP remains isolated following detailed prenatal imaging, genetic evaluation, and postnatal assessment. By contrast, complex ASP and ASP associated with suspected or confirmed SOD require cautious counseling and comprehensive multidisciplinary care. The descriptive pooled values reported in this review should be used to inform counseling only as approximate observations derived from heterogeneous retrospective data, not as precise quantitative risk estimates.

Fetal MRI is a valuable adjunct to prenatal US and improves characterization of associated CNS anomalies, but it cannot reliably exclude SOD. Genetic testing may identify underlying etiologies in selected cases, although its role requires further standardization. Long-term follow-up by specialists in fetal medicine, neuroradiology, genetics, pediatric neurology, ophthalmology, and endocrinology is essential because clinically relevant complications may emerge after birth [29]. Larger prospective studies with standardized imaging protocols, uniform definitions of isolated ASP, systematic genetic testing, formal risk-of-bias reporting, and extended neurodevelopmental follow-up are needed to improve prognostic accuracy and parental counseling.

## Figures and Tables

**Table 1:** Characteristics and main findings of studies included in the structured narrative review.

Study	Prenatal Sample and Classification	Assessment and Follow-Up	Main Findings
Pickup et al. [23]	35 fetuses; 17 prenatally isolated ASP, 18 complex ASP.	Fetal MRI; ACC excluded; genetic or prenatal screening abnormalities in selected tested cases. Postnatal reclassification and clinical follow-up were available for part of the cohort.	Only 5/17 prenatally isolated cases remained isolated; complex ASP was associated with developmental delay, seizures, hydrocephalus, abnormal tone, feeding problems, and sensory abnormalities.
Vawter-Lee et al. [24]	8 fetuses with fetal MRI diagnosis of ASP with or without VMG and no additional prenatal abnormalities.	All underwent fetal MRI; ventricular size ranged from normal to severe VMG. Postnatal MRI, ophthalmological, and endocrine assessment were performed in all cases.	Postnatal MRI showed isolated ASP in 4/8 and complex findings in 4/8; two children were diagnosed with SOD.
Borkowski-Tillman et al. [25]	47 fetuses with prenatal US or MRI diagnosis of partial or complete ASP; 17 isolated and 30 complex.	MRI was performed in 29/47; US and MRI were concordant in most cases; genetic testing was usually normal when performed. Postnatal outcome data were available for most delivered isolated cases; TOP was more common in complex cases.	Fourteen delivered isolated cases showed normal development; complex cases had more variable neurological and syndromic outcomes.
Shinar et al. [21]	214 ASP cases; analysis emphasized 18 cases with isolated ASP suspected of SOD.	MRI in 11/18; optic chiasm and related structures assessed when visible; one VUS was reported. Live-born infants underwent postnatal follow-up, including assessment for SOD.	Among followed live-born infants, five were diagnosed with SOD and five were not; SOD cases had more visual, growth, seizure, and developmental morbidity.
Pilliod et al. [26]	15 fetuses with isolated ASP; VMG < 15 mm was allowed if no other intracranial abnormality was present.	MRI was performed in 11/15 and confirmed US diagnosis; genetic testing was performed in 10/15. Twelve infants were live-born; postnatal imaging was available for 10 infants.	Outcomes ranged from normal development to mild motor findings and significant visual/endocrine involvement in optic nerve hypoplasia cases.
Di Pasquo et al. [20]	15 fetuses with apparently isolated ASP and available postnatal follow-up.	Prenatal US and/or MRI; genetic testing was normal in all tested cases; optic chiasm evaluation was reported. Postnatal follow-up was available for all included cases.	Fourteen children had normal development; one child had SOD with visual impairment. Only original cohort data were extracted from this cohort/meta-analysis publication.
Viñals et al. [27]	8 ASP cases compared with 115 morphologically normal fetuses for optic chiasm measurement.	MRI was performed in 4/8 ASP cases; optic chiasm width was measured by two-dimensional US. Postnatal visual and clinical outcomes were reported.	All five isolated ASP cases had normal vision; complex ASP with small optic chiasm was associated with SOD and neurological morbidity.

ACC, agenesis of the corpus callosum; ASP, absent septum pellucidum; MRI, magnetic resonance imaging; SOD, septo-optic dysplasia; TOP, termination of pregnancy; US, ultrasonography; VMG, ventriculomegaly.

**Table 2:** Genetic work-up and reported diagnostic yield across included studies.

Study	ASP Group Tested	No. Tested/Eligible	Testing Modality	Abnormal Findings	VUS	Interpretation
Pickup et al. [23]	Prenatally isolated and complex ASP	21 tested/35 total (reported as genetic or prenatal screening abnormalities)	Genetic testing or prenatal screening; exact modalities not uniformly specified	5/21 abnormal genetic or prenatal screening results; one intrauterine fetal demise reported	NR	Yield by modality not calculable; abnormalities occurred in selected tested cases.
Vawter-Lee et al. [24]	ASP with or without VMG and no additional prenatal abnormality	NR/8	Not uniformly reported	NR	NR	Insufficient data to estimate genetic yield.
Borkowski-Tillman et al. [25]	Isolated ASP; complex ASP with CNS or non-CNS findings	13 tested/47 total: 2/17 isolated; 7/24 complex CNS; 4/6 complex non-CNS	Karyotype and/or CMA as reported	One chromosome X microdeletion in complex ASP; otherwise normal among tested cases	NR	Low apparent yield in isolated ASP; limited by selective testing and small denominators.
Shinar et al. [21]	Isolated ASP suspected of SOD	9/18	Karyotype/CMA as reported	No pathogenic abnormality reported	1 VUS in chromosome 10p13 in a postnatally confirmed SOD case	Yield uncertain; cohort enriched for suspected SOD.
Pilliod et al. [26]	Isolated absent CSP/ASP, mild VMG allowed if no other intracranial abnormality	9/15	2 CMA; 7 karyotype	One 30-kb deletion at 1p14 in a pregnancy ending in TOP; 8/9 normal	NR	Low apparent yield; very small CMA denominator.
Di Pasquo et al. [20]	Apparently isolated ASP with postnatal follow-up	11/15	Karyotype and/or CMA as reported	All tested cases normal	NR	Low apparent yield in tested apparently isolated ASP cases.
Viñals et al. [27]	Isolated and complex ASP cases used for optic chiasm assessment	NR/8	NR	NR	NR	Not estimable.

ASP, absent septum pellucidum; CMA, chromosomal microarray analysis; CSP, cavum septi pellucidi; NR, not reported; SOD, septo-optic dysplasia; TOP, termination of pregnancy; VUS, variant of uncertain significance; VMG, ventriculomegaly.

## Data Availability

No new data were created in this study. The data extracted and synthesized in this review are available in the cited publications and summarized in the tables of this manuscript.

## Supplementary Materials

The following supporting information can be downloaded at: https://www.mdpi.com/article/10.3390/jcm15134889/s1, PRISMA 2020 checklist [22]; Table S1: detailed characteristics of included studies; Table S2: full electronic search strategy and updated search; Table S3: methodological quality assessment using Joanna Briggs Institute critical appraisal tools; Table S4: genetic work-up across included studies; Table S5: postnatal follow-up and neurodevelopmental assessment across included studies. All references cited in the Supplementary Materials are included in the main reference list.
