# Supplementary material for: Absent Septum Pellucidum in Fetal Development: Diagnostic Challenges, Associated Anomalies, and Prognostic Uncertainty—A Structured Narrative Review
Source: J Clin Med. 2026 Jun 23;15(13):4889. doi: 10.3390/jcm15134889 (PMC13362233; doi:10.3390/jcm15134889)
Supplement: Supplementary file 1 [file jcm-15-04889-s001.zip › jcm-4336729-PRISMA.pdf]

PRISMA 2020 Checklist

Section and Topic	Item #	Checklist item	Location where item is reported
**TITLE**			
Title	1	Identify the report as a systematic review.	Title page: manuscript title identifies the article as "A Systematic Review".
**ABSTRACT**			
Abstract	2	See the PRISMA 2020 for Abstracts checklist.	Abstract: structured abstract with Background/Objectives, Methods, Results, and Conclusions.
**INTRODUCTION**			
Rationale	3	Describe the rationale for the review in the context of existing knowledge.	Introduction: paragraphs describing ASP/CSP background, diagnostic uncertainty, associated anomalies, SOD risk, and existing evidence.
Objectives	4	Provide an explicit statement of the objective(s) or question(s) the review addresses.	Introduction: final paragraph states the objective of synthesizing evidence on prenatal imaging, associated anomalies, genetic evaluation, and postnatal outcomes.
**METHODS**			
Eligibility criteria	5	Specify the inclusion and exclusion criteria for the review and how studies were grouped for the syntheses.	Materials and Methods: eligibility and exclusion criteria; Results/Table 1: grouping as prenatal isolated ASP, complex ASP, postnatally isolated ASP, postnatally complex ASP, and SOD-related outcomes.
Information sources	6	Specify all databases, registers, websites, organisations, reference lists and other sources searched or consulted to identify studies. Specify the date when each source was last searched or consulted.	Materials and Methods: PubMed and Google Scholar, reference-list screening, publication years 2014-2024, and search date of 5 November 2024.
Search strategy	7	Present the full search strategies for all databases, registers and websites, including any filters and limits used.	Materials and Methods: search terms reported for PubMed/Google Scholar, including "absent septum pellucidum" combined with "fetal MRI", "prenatal ultrasound", "holoprosencephaly", and "septo-optic dysplasia"; English/full-text limits

PRISMA

PRISMA 2020 Checklist

Section and Topic	Item #	Checklist item	Location where item is reported
			reported.
Selection process	8	Specify the methods used to decide whether a study met the inclusion criteria of the review, including how many reviewers screened each record and each report retrieved, whether they worked independently, and if applicable, details of automation tools used in the process.	Materials and Methods and Figure 1: eligibility decisions and numbers in the study-selection flow are reported. Number of reviewers, independent screening, and automation tools are not reported.
Data collection process	9	Specify the methods used to collect data from reports, including how many reviewers collected data from each report, whether they worked independently, any processes for obtaining or confirming data from study investigators, and if applicable, details of automation tools used in the process.	Materials and Methods: data extraction using a standardized form is reported. Number of reviewers, independent extraction, investigator contact, and automation tools are not reported.
Data items	10a	List and define all outcomes for which data were sought. Specify whether all results that were compatible with each outcome domain in each study were sought (e.g. for all measures, time points, analyses), and if not, the methods used to decide which results to collect.	Materials and Methods: outcomes sought included postnatal imaging, ophthalmological and endocrine evaluation, neurodevelopmental outcomes, TOP, and perinatal outcomes; Results/Tables 3-4 present outcome data.
	10b	List and define all other variables for which data were sought (e.g. participant and intervention characteristics, funding sources). Describe any assumptions made about any missing or unclear information.	Materials and Methods: other variables included study design, year, country/referral setting, sample size, inclusion/exclusion criteria, gestational age, imaging modality, isolated/complex ASP classification, associated anomalies, and genetic testing results.
Study risk of bias assessment	11	Specify the methods used to assess risk of bias in the included studies, including details of the tool(s) used, how many reviewers assessed each study and whether they worked independently, and if applicable, details of automation tools used in the process.	Not reported: no formal study risk-of-bias assessment method or tool is described.
Effect measures	12	Specify for each outcome the effect measure(s) (e.g. risk ratio, mean difference) used in the synthesis or presentation of results.	Results/Tables 3-4: descriptive counts and percentages are presented. No comparative effect measures (e.g., risk ratios or mean differences) are specified.
Synthesis methods	13a	Describe the processes used to decide which studies were eligible for each synthesis (e.g. tabulating the study intervention characteristics and comparing against the planned groups for each synthesis (item #5)).	Materials and Methods: studies eligible for descriptive synthesis were selected according to

Section and Topic	Item #	Checklist item	Location where item is reported
			prenatal diagnosis, associated anomalies/genetic findings, and outcome reporting; Table 1 tabulates study characteristics.
	13b	Describe any methods required to prepare the data for presentation or synthesis, such as handling of missing summary statistics, or data conversions.	Materials and Methods and Results: data were extracted into standardized categories and summarized descriptively; no missing-statistic imputation or numerical conversions are reported.
	13c	Describe any methods used to tabulate or visually display results of individual studies and syntheses.	Figure 1 and Tables 1-4: flow diagram, study-characteristics table, genetic findings table, developmental-outcome table, and SOD-risk table.
	13d	Describe any methods used to synthesize results and provide a rationale for the choice(s). If meta-analysis was performed, describe the model(s), method(s) to identify the presence and extent of statistical heterogeneity, and software package(s) used.	Materials and Methods/Results: narrative and descriptive synthesis using counts and percentages; no meta-analysis was performed.
	13e	Describe any methods used to explore possible causes of heterogeneity among study results (e.g. subgroup analysis, meta-regression).	Discussion/Strengths and Limitations: heterogeneity in definitions of isolated ASP, imaging protocols, genetic testing, and follow-up is discussed; no formal subgroup analysis or meta-regression was performed.
	13f	Describe any sensitivity analyses conducted to assess robustness of the synthesized results.	Not applicable/not reported: no sensitivity analyses were conducted or reported.
Reporting bias assessment	14	Describe any methods used to assess risk of bias due to missing results in a synthesis (arising from reporting biases).	Not reported: no assessment of reporting bias or missing-results bias is described.
Certainty assessment	15	Describe any methods used to assess certainty (or confidence) in the body of evidence for an outcome.	Not reported: no formal certainty-of-evidence assessment (e.g., GRADE) is described.
**RESULTS**			
Study selection	16a	Describe the results of the search and selection process, from the number of records identified in the search to the number of studies included in the review, ideally using a flow diagram.	Results: study-selection numbers from records identified to studies included; Figure 1: PRISMA 2020 flow diagram.

PRISMA 2020 Checklist

Section and Topic	Item #	Checklist item	Location where item is reported
	16b	Cite studies that might appear to meet the inclusion criteria, but which were excluded, and explain why they were excluded.	Results/Figure 1: exclusion categories and counts are reported. Individual excluded studies that might appear eligible are not cited.
Study characteristics	17	Cite each included study and present its characteristics.	Results/Table 1 and References: each included study is cited and its key characteristics are summarized.
Risk of bias in studies	18	Present assessments of risk of bias for each included study.	Not reported: risk-of-bias assessments for individual studies are not presented.
Results of individual studies	19	For all outcomes, present, for each study: (a) summary statistics for each group (where appropriate) and (b) an effect estimate and its precision (e.g. confidence/credible interval), ideally using structured tables or plots.	Results/Table 1: individual study findings; Tables 3-4: outcome counts. Effect estimates with precision intervals are not presented because synthesis is descriptive.
Results of syntheses	20a	For each synthesis, briefly summarise the characteristics and risk of bias among contributing studies.	Results sections 3.1-3.4 and Tables 1-4 summarize contributing study characteristics and descriptive findings. Risk of bias among contributing studies is not formally assessed.
	20b	Present results of all statistical syntheses conducted. If meta-analysis was done, present for each the summary estimate and its precision (e.g. confidence/credible interval) and measures of statistical heterogeneity. If comparing groups, describe the direction of the effect.	Not applicable: no statistical synthesis/meta-analysis was conducted; descriptive counts and percentages are reported in Results and Tables 3-4.
	20c	Present results of all investigations of possible causes of heterogeneity among study results.	Discussion/Strengths and Limitations: possible heterogeneity sources are discussed qualitatively; no formal heterogeneity investigation is reported.
	20d	Present results of all sensitivity analyses conducted to assess the robustness of the synthesized results.	Not applicable: no sensitivity analyses were conducted.
Reporting biases	21	Present assessments of risk of bias due to missing results (arising from reporting biases) for each synthesis assessed.	Not reported: no assessment of reporting bias due to missing results is presented.
Certainty of evidence	22	Present assessments of certainty (or confidence) in the body of evidence for each outcome assessed.	Not reported: no certainty-of-evidence assessment is presented.
**DISCUSSION**			

PRISMA 2020 Checklist

Section and Topic	Item #	Checklist item	Location where item is reported
Discussion	23a	Provide a general interpretation of the results in the context of other evidence.	Discussion: interpretation of diagnostic and prognostic heterogeneity of ASP in the context of prenatal imaging, SOD, complex ASP, and previous evidence.
	23b	Discuss any limitations of the evidence included in the review.	Discussion/Strengths and Limitations: limitations of included evidence, including small samples, heterogeneous imaging protocols, inconsistent genetic testing, variable follow-up, and TOP-related outcome limitations.
	23c	Discuss any limitations of the review processes used.	Materials and Methods and Discussion/Strengths and Limitations: limitations of review processes include small number of studies, heterogeneity of definitions, incomplete follow-up, and inability to perform formal meta-analysis.
	23d	Discuss implications of the results for practice, policy, and future research.	Discussion and Conclusions: implications for prenatal counselling, multidisciplinary follow-up, genetic evaluation, postnatal ophthalmological/endocrine assessment, and future prospective studies.
**OTHER INFORMATION**			
Registration and protocol	24a	Provide registration information for the review, including register name and registration number, or state that the review was not registered.	Materials and Methods: the review was not preregistered.
	24b	Indicate where the review protocol can be accessed, or state that a protocol was not prepared.	Not reported: protocol availability is not stated.
	24c	Describe and explain any amendments to information provided at registration or in the protocol.	Not applicable: no registration/protocol amendments are applicable because the review was not preregistered and no protocol is reported.
Support	25	Describe sources of financial or non-financial support for the review, and the role of the funders or sponsors in the review.	Funding: this research received no external funding.
Competing	26	Declare any competing interests of review authors.	Conflicts of Interest: the authors declare no conflict

PRISMA 2020 Checklist

Section and Topic	Item #	Checklist item	Location where item is reported
interests			of interest.
Availability of data, code and other materials	27	Report which of the following are publicly available and where they can be found: template data collection forms; data extracted from included studies; data used for all analyses; analytic code; any other materials used in the review.	Data Availability Statement: no new data were created; extracted and synthesized data are available in cited publications and summarized in manuscript tables. No analytic code is reported.

From: Page MJ, McKenzie JE, Bossuyt PM, Boutron I, Hoffmann TC, Mulrow CD, et al. The PRISMA 2020 statement: an updated guideline for reporting systematic reviews. BMJ 2021;372:n71. doi: 10.1136/bmj.n71. This work is licensed under CC BY 4.0. To view a copy of this license, visit https://creativecommons.org/licenses/by/4.0/
