# Supplementary material for: Absent Septum Pellucidum in Fetal Development: Diagnostic Challenges, Associated Anomalies, and Prognostic Uncertainty—A Structured Narrative Review
Source: J Clin Med. 2026 Jun 23;15(13):4889. doi: 10.3390/jcm15134889 (PMC13362233; doi:10.3390/jcm15134889)
Supplement: Supplementary file 1 [file jcm-15-04889-s001.zip › jcm-4336729-supplementary.pdf]

Supplementary Material

Absent Septum Pellucidum in Fetal Development: Diagnostic Challenges, Associated Anomalies, and Prognostic Uncertainty - A Structured Narrative Review

Supplementary Table S1. Detailed characteristics of included studies

Study	Sample/classification	Imaging/genetics	Postnatal assessment/follow-up	Key outcomes
Pickup et al. [22]	35 fetuses; 17 prenatally isolated ASP, 18 complex ASP.	Fetal MRI; ACC excluded; genetic or prenatal screening abnormalities in selected tested cases.	Postnatal reclassification and clinical follow-up were available for part of the cohort.	Only 5/17 prenatally isolated cases remained isolated; complex ASP was associated with developmental delay, seizures, hydrocephalus, abnormal tone, feeding problems, and sensory abnormalities.
Vawter-Lee et al. [23]	8 fetuses with fetal MRI diagnosis of ASP with or without VMG and no additional prenatal abnormalities.	All underwent fetal MRI; ventricular size ranged from normal to severe VMG.	Postnatal MRI, ophthalmological, and endocrine assessment were performed in all cases.	Postnatal MRI showed isolated ASP in 4/8 and complex findings in 4/8; two children were diagnosed with SOD.
Borkowski-Tillman et al. [24]	47 fetuses with prenatal US or MRI diagnosis of partial or complete ASP; 17 isolated and 30 complex.	MRI was performed in 29/47; US and MRI were concordant in most cases; genetic testing was usually normal when performed.	Postnatal outcome data were available for most delivered isolated cases; TOP was more common in complex cases.	Fourteen delivered isolated cases showed normal development; complex cases had more variable neurological and syndromic outcomes.
Shinar et al. [21]	214 ASP cases; analysis emphasized 18 cases with isolated ASP suspected of SOD.	MRI in 11/18; optic chiasm and related structures assessed when visible; one VUS was reported.	Live-born infants underwent postnatal follow-up, including assessment for SOD.	Among followed live-born infants, five were diagnosed with SOD and five were not; SOD cases had more visual, growth, seizure, and developmental morbidity.
Pilliod et al. [25]	15 fetuses with isolated ASP; VMG <15 mm was allowed if no other intracranial abnormality was present.	MRI was performed in 11/15 and confirmed US diagnosis; genetic testing was performed in 10/15.	Twelve infants were live-born; postnatal imaging was available for 10 infants.	Outcomes ranged from normal development to mild motor findings and significant visual/endocrine involvement in optic nerve hypoplasia cases.
Di Pasquo et al. [20]	15 fetuses with apparently isolated ASP and available postnatal follow-up.	Prenatal US and/or MRI; genetic testing was normal in all tested cases; optic chiasm evaluation was reported.	Postnatal follow-up was available for all included cases.	Fourteen children had normal development; one child had SOD with visual impairment. Only original cohort data were extracted from this cohort/meta-analysis publication.

Viñals et al. [26]	8 ASP cases compared with 115 morphologically normal fetuses for optic chiasm measurement.	MRI was performed in 4/8 ASP cases; optic chiasm width was measured by two-dimensional US.	Postnatal visual and clinical outcomes were reported.	All five isolated ASP cases had normal vision; complex ASP with small optic chiasm was associated with SOD and neurological morbidity.

Supplementary Table S2. Full electronic search strategy

Date of initial search: 5 November 2024. Updated search: 8 June 2026. Limits: English-language full text; publication years 2014 through the updated search date; human fetal/prenatal studies when filters were available. The updated search used the same sources and search strings and did not identify additional eligible primary prenatal ASP outcome cohorts. Final search verification confirmed that all eligible primary prenatal ASP outcome cohorts found through 8 June 2026 are included in Supplementary Table S1 and cited in the manuscript where appropriate.

Database/source	Search string	Notes
PubMed	("absent septum pellucidum"[Title/Abstract] OR "absent cavum septi pellucidi"[Title/Abstract] OR "agenesis of septum pellucidum"[Title/Abstract]) AND ("fetal MRI"[Title/Abstract] OR "prenatal ultrasound"[Title/Abstract] OR prenatal[Title/Abstract] OR fetal[Title/Abstract])	Primary bibliographic database searched.
PubMed	"absent septum pellucidum" AND "fetal MRI"	Free-text search used to maximize retrieval.
PubMed	"absent septum pellucidum" AND "prenatal ultrasound"	Free-text search used to maximize retrieval.
PubMed	"absent septum pellucidum" AND "holoprosencephaly"	Used for differential-diagnosis retrieval.
PubMed	"absent septum pellucidum" AND "septo-optic dysplasia"	Used for SOD-related retrieval.
Google Scholar	"absent septum pellucidum" "fetal MRI"; "absent septum pellucidum" "prenatal ultrasound"; "absent septum pellucidum" "septo-optic dysplasia"; "absent cavum septi pellucidi" prenatal	First relevant results were screened, followed by manual screening of reference lists of relevant studies and reviews.
PubMed and Google Scholar update	Same search strings as above, repeated on 8 June 2026	Updated search performed to address reviewer request; no additional eligible primary prenatal ASP outcome cohorts were identified. All eligible primary prenatal ASP outcome cohorts found by the final search were included in Supplementary Table S1 and cited in the manuscript where appropriate. Recent narrative/practical recommendations were used only for contextual discussion where relevant.

Supplementary Table S3. Methodological quality assessment

Methodological quality was appraised with Joanna Briggs Institute critical appraisal tools for cohort and case series studies, as appropriate. This qualitative summary was used to contextualize the certainty of descriptive findings rather than to exclude studies.

Study	Design/tool applied	Main strengths	Main limitations/risk-of-bias concerns	Overall appraisal
Pickup et al. [22]	Retrospective cohort; JBI cohort/case series domains	Clear fetal MRI-based classification; clinically relevant postnatal outcomes.	Retrospective design, limited sample size, incomplete postnatal confirmation in some cases, variable follow-up.	Moderate risk of bias.
Vawter-Lee et al. [23]	Small retrospective cohort/case series; JBI case series domains	All cases underwent fetal MRI and postnatal ophthalmological/endocrine assessment.	Very small sample size; heterogeneous VMG severity; limited generalizability.	Moderate to high risk of bias.
Borkowski-Tillman et al. [24]	Retrospective cohort; JBI cohort domains	Larger sample than most included studies; distinction between isolated and complex ASP.	Retrospective referral-center design; TOP and loss to follow-up may bias outcome estimates.	Moderate risk of bias.
Shinar et al. [21]	Retrospective cohort/subgroup analysis; JBI cohort domains	Detailed SOD-focused postnatal outcome assessment.	Subgroup enrichment for suspected SOD; not fully representative of all isolated ASP cases.	Moderate risk of bias.
Pilliod et al. [25]	Retrospective cohort/case series; JBI case series domains	Prenatal MRI confirmation in most cases; genetic testing reported in many cases.	Small sample; mild VMG allowed within isolated group; follow-up incomplete in some domains.	Moderate risk of bias.
Di Pasquo et al. [20]	Original cohort plus meta-analysis; JBI cohort domains applied to original cohort	All included cohort cases had postnatal follow-up; relevant optic pathway assessment.	Small original cohort; meta-analysis component not used as primary data here to avoid duplication.	Low to moderate risk of bias.
Viñals et al. [26]	Case series with comparison group for optic chiasm measurement; JBI case series domains	Objective optic chiasm measurement and visual outcome reporting.	Small number of ASP cases; complex and isolated cases combined for some imaging comparisons.	Moderate risk of bias.

Abbreviations: ACC, agenesis of the corpus callosum; ASP, absent septum pellucidum; CMA, chromosomal microarray analysis; CNS, central nervous system; CSP, cavum septi pellucidi; JBI, Joanna Briggs Institute; MRI, magnetic resonance imaging; NR, not reported; SOD, septo-optic dysplasia; TOP, termination of pregnancy; VUS, variant of uncertain significance; VMG, ventriculomegaly.

Supplementary Table S4. Genetic work-up across included studies

NR denotes not reported. Because testing indications, denominators, and modalities differed substantially across studies, diagnostic yield could not be pooled by modality.

**Study**	**ASP group tested**	**No. tested / eligible**	**Testing modality**	**Abnormal findings**	**VUS**	**Interpretation**
Pickup et al. [22]	Prenatally isolated and complex ASP	21 tested / 35 total (reported as genetic or prenatal	Genetic testing or prenatal screening; exact modalities not	5/21 abnormal genetic or prenatal screening results; one	NR	Yield by modality not calculable; abnormalities occurred in

Study	ASP group tested	No. tested / eligible	Testing modality	Abnormal findings	VUS	Interpretation
		screening abnormalities)	uniformly specified	intrauterine fetal demise reported		selected tested cases.
Vawter-Lee et al. [23]	ASP with or without VMG and no additional prenatal abnormality	NR / 8	Not uniformly reported	NR	NR	Insufficient data to estimate genetic yield.
Borkowski-Tillman et al. [24]	Isolated ASP; complex ASP with CNS or non-CNS findings	13 tested / 47 total: 2/17 isolated; 7/24 complex CNS; 4/6 complex non-CNS	Karyotype and/or CMA as reported	One chromosome X microdeletion in complex ASP; otherwise normal among tested cases	NR	Low apparent yield in isolated ASP; limited by selective testing and small denominators.
Shinar et al. [21]	Isolated ASP suspected of SOD	9/18	Karyotype/CMA as reported	No pathogenic abnormality reported	1 VUS in chromosome 10p13 in a postnatally confirmed SOD case	Yield uncertain; cohort enriched for suspected SOD.
Pilliod et al. [25]	Isolated absent CSP/ASP, mild VMG allowed if no other intracranial abnormality	9/15	2 CMA; 7 karyotype	One 30-kb deletion at 1p14 in a pregnancy ending in TOP; 8/9 normal	NR	Low apparent yield; very small CMA denominator.
Di Pasquo et al. [20]	Apparently isolated ASP with postnatal follow-up	11/15	Karyotype and/or CMA as reported	All tested cases normal	NR	Low apparent yield in tested apparently isolated ASP cases.
Viñals et al. [26]	Isolated and complex ASP cases used for optic chiasm assessment	NR / 8	NR	NR	NR	Not estimable.

Supplementary Table S5. Detailed postnatal follow-up characteristics and neurodevelopmental assessment across included studies

Study	Follow-up completeness	Age/duration of follow-up	Postnatal imaging	Ophthalmological/endocrine assessment	Developmental/neurological assessment	Key limitations for outcome interpretation
Pickup et al. [22]	Follow-up was available for 22/33 live-born infants. Postnatal evaluation was available for 10/17 prenatally isolated ASP cases; 7/17 prenatally isolated and 6/18 prenatally complex cases were lost to follow-up.	Mean interval from first to most recent postnatal visit: 520 ± 515 days; age range at follow-up: 0-1907 days.	Twenty infants had at least one postnatal brain MRI and two had head ultrasound only. Mean age at first postnatal imaging was 45.6 +/- 87.4 days.	Postnatal specialty assessments included endocrinology and ophthalmology in selected cases; assessments were not uniformly available for all live-born infants.	Assessments included neurology, developmental pediatrics, endocrinology, ophthalmology and genetics. Fourteen of twenty followed infants had three or more postnatal specialty assessments.	Retrospective single-center design; incomplete postnatal evaluation; developmental data derived from clinical records rather than uniform prospective testing.
Vawter-Lee et al. [23]	All 8/8 included infants completed postnatal MRI, endocrine evaluation and ophthalmological evaluation.	Follow-up range: 8-72 months. Developmental screening age ranged from 8 months to 6 years, with a mean of 28 months.	Postnatal brain MRI was performed in all eight infants, between 1 day and 4 months of age.	Postnatal ophthalmological examinations were performed, usually on days 2-3 of life. Endocrine evaluation included neonatal pituitary-function testing, including thyroid function and ACTH/cortisol-axis assessment where available.	Development was assessed with standardized tools: ASQ-3 for children aged 1 month to 5.5 years and PEDS for children aged 5.5-8 years. Seven of eight children had normal developmental screening.	Very small sample; selected cohort requiring complete postnatal evaluation; no fully standardized endocrine protocol.

Borkowski-Tillman et al. [24]	Isolated ASP: follow-up available for all 14 live-born children; three isolated cases were lost to follow-up before delivery/outcome assessment. CNS-associated ASP: 13 live-born children, of whom two were lost to follow-up.	Isolated ASP: mean follow-up 45 months (range, 3-84 months). CNS-associated ASP with normal development: mean follow-up 21 months (range, 1-42 months).	Postnatal imaging after delivery was recorded when available and compared with prenatal findings.	Recorded postnatal data included eyesight problems, blindness or optic-disc hypoplasia, endocrine test results and clinical neurological examinations.	Clinical neurological examinations were recorded according to local center protocols. All 14 delivered isolated ASP cases developed normally.	Retrospective multicenter design; variable center-specific protocols; outcome assessment affected by TOP and loss to follow-up, particularly in complex cases.
Shinar et al. [21]	Follow-up was available for live-born infants from the subgroup with prenatally suspected SOD; exact completeness should be interpreted according to the original cohort structure.	Follow-up in the SOD-focused cohort extended into childhood; detailed duration varied across cases.	Prenatal MRI was performed in selected cases; postnatal assessment was used to determine whether SOD was present.	Postnatal follow-up focused on SOD-related morbidity, including visual and endocrine outcomes.	Reported outcomes included visual impairment, growth/endocrine concerns, seizures and developmental morbidity.	Subgroup enriched for suspected SOD; therefore, results are not representative of all apparently isolated ASP cases.
Pilliod et al. [25]	Fifteen prenatally diagnosed isolated absent CSP cases were identified. Twelve pregnancies continued; ten surviving infants were discharged alive. Long-term follow-up was available for eight surviving children; two families declined postnatal imaging.	Follow-up duration varied and was not standardized. One SOD case was followed through 2 years of age; some follow-up was limited to early infancy.	Postnatal imaging was available in ten cases. Absent CSP was confirmed in all but one discordant case; two children did not undergo postnatal imaging.	Pituitary function was evaluated in all ten surviving neonates. Dilated-eye examination was performed and showed two cases of hypoplastic optic nerves, two cases of retinopathy of prematurity and six normal examinations.	Clinical outcomes were abstracted from medical records. Neurological and developmental follow-up were not standardized by assessment type, provider or age at assessment.	Incomplete and heterogeneous long-term follow-up; two neonatal deaths related to extreme prematurity; clinical assessment not prospectively standardized.
Di Pasquo et al. [20]	Original cohort: 18 apparently isolated ASP cases; three were lost to follow-up and 15 were included in the final cohort analysis.	Median postnatal follow-up: 36 months (range, 12-60 months).	Pre- and postnatal US and MRI findings were collected; postnatal discordant or additional imaging findings were assessed.	Follow-up data included ophthalmological assessment and endocrine dysfunction. Optic pathways were assessed antenatally in most cases.	Data were collected from infant medical notes or by telephone questionnaire addressed to parents and/or the family pediatrician. Neurological outcomes included motor deficit, epilepsy, intellectual disability, language delay, learning difficulties and behavioral disorders.	Small original cohort; some follow-up was questionnaire-based; meta-analysis component was not used as primary data in the present review to avoid duplication.
Viñals et al. [26]	Complete follow-up was available for all eight fetuses with agenesis of the SP.	Follow-up ranged from 6 months to 7 years; individual ages were reported in the study table.	Fetal MRI was available in selected cases; optic chiasm width was assessed by two-dimensional neurosonography.	Postnatal ophthalmological examination was available in all cases. Clinical signs of anterior or posterior hypopituitarism were reported as absent in cases with normal optic chiasm width.	Clinical outcome included vision, nystagmus, epilepsy, psychomotor delay, hearing abnormality and developmental status.	Small case series designed primarily to assess optic chiasm measurement rather than standardized developmental testing; no uniform formal endocrine protocol reported.

ASP, absent septum pellucidum; ACTH, adrenocorticotropic hormone; ASQ-3, Ages and Stages Questionnaire, Third Edition; CSP, cavum septi pellucidi; MRI, magnetic resonance imaging; PEDS, Parents' Evaluation of Developmental Status; SOD, septo-optic dysplasia; TOP, termination of pregnancy; US, ultrasound. Follow-up methods, age at assessment and completeness varied substantially across studies; therefore, pooled developmental outcomes should be interpreted as descriptive observations rather than precise prognostic estimates.
